# Clinical Society of London—Conclusions of the Myxœdema Committee

**Published:** 1888-09

**Authors:** 


### CLINICAL SOCIETY OF LONDON—CONCLUSIONS OF THE MYXŒDEMA COMMITTEE.

Dr. Ord, as the chairman of the myxœdema committee read the following conclusions. He admitted that the time taken up in the investigation was protracted, but he claimed that when the results were known the time would not appear out of proportion.

1. That myxœdema is a well defined disease.

2. That the disease affects women much more frequently than men, and that the subjects are, for the most part, of middle age.

3. That clinical and pathological observations respectively indicate, in a decisive way, that the one condition, common to all cases, is destructive change of the thyroid gland.

4. That the most common form of destructive change of the thyroid gland consists in the substitution of a delicate fibrous tissue for the proper glandular structure.

5. That interstitial development of fibrous tissue is also observed very frequently in the skin, and with much less frequency in the viscera, the appearances presented by this tissue being suggestive of an irritative or inflammatory process.

6. That pathological observation, while showing cause for the changes in the skin during life, for the falling off of the hair and the loss of the teeth, for the increased bulk of the body, as due to the excess of subcutaneous fat, affords no explanation of the affections of speech, movement, sensation, consciousness, and intellect, which form a large part of the symptoms of the disease.

7. That chemical examination of the comparatively few available cases fails to show the general existence of an excess of mucin in the tissues adequately corresponding to the amount recorded in the first observations, but that this discrepancy may be, in part, attributed to the fact that tumefaction of the integuments, although generally characteristic of myxœdema, varies considerably throughout the course of the disease, and often disappears shortly before death.

8. That in experiments made upon animals, particularly on monkeys, symptoms resembling, in a very close and remarkable way, those of myxœdema have followed complete removal of the thyroid gland, performed under antiseptic precautions, and with, as far as could be ascertained, no injury to the adjacent nerves, or to the trachea.

9. That in such experimental cases, a large excess of mucin has been found to be present in the skin, fibrous tissues, blood and salivary glands; in particular, the parotid gland, normally containing no mucin, has presented that substance in quantities corresponding to what would be ordinarily found in the submaxillary gland.

10. That the full analysis of the results of the removal of the thyroid gland in man demonstrates in an important proportion of the cases the fact of the subsequent development of symptoms exactly corresponding with those of myxœdema.

11. That, in no inconsiderable number of cases, the operation has not been followed by such symptoms, the apparent immunity being in many cases probably due to the presence and subsequent development of accessory thyroid glands, or accidentally incomplete removal, or to insufficiently long observation of the patients after operation.

12. That whereas injury to the trachea, atrophy of the trachea, injury of the recurrent larngeal nerves, injury of the cervical sympathetic, and endemic influences have been, by various observers, supposed to be the true causes of experimental or of operative myxœdema (cahexia strumipriva), there is, in the first place, no evidence to show that, of the numerous and various surgical operations performed on the neck and throat, involving various organs and tissues, none, save those in which the thyroid gland has been removed, have been followed by the symptoms under consideration; that in many of the operations on men, and in most, if not all, of the experimental operations made by Professor Horsley on monkeys and other animals, the procedure avoided all injury of surrounding parts, and was perfectly aseptic; that myxœdema has followed removal of the thyroid gland in persons neither living in, nor having lived in, localities, the seat of endemic cretinism; that therefore, the positive evidence on this point outweighs vastly the negative, and that it appears strongly proved that myxœdema is frequently produced by the removal, as well as by the pathological destruction, of the thyroid gland.

13. That whereas, according to clause 2, in myxœdema women are much more numerously affected than men, in the operative form of myxœdema no important difference of the same kind is observed.

14. That a general review of symptoms and pathology leads to the belief that the disease described under the name of myxœdema, as observed in adults, is practically the same disease as that named sporadic cretinism, when affecting children; that myxœdema is probably identical with cachexia strumipriva; and that a very close affinity exists between myxœdema and endemic cretinism.

15. That while these several conditions appear, in the main, to depend on, or to be associated with, destruction or loss of the function of the thyroid gland, the ultimate cause of such destruction or loss is at present not evident.

The president (Dr. Broadbent) congratulated the society on the completion of this important investigation, and said their thanks were due to the committee for the enormous work and to the zeal and abilities which had enabled them to present this summary of the report, and practically the report itself, during the present session. When they heard the conclusions and imagined the enormous amount of investigation and research and inquiry which was embodied there, four years was a comparatively short period. The report would make the present session memorable. It reflected the greatest possible credit upon the society. It was the most important work yet carried out in its name. Already the labors of the committee had served to stimulate the carrying out of similar investigations in Germany. He then concluded with a graceful tribute to the way in which Dr. Ord's name was indissolubly bound up with the history of myxœdema.—*Medical Press and Circular.*

